# The Corn Sweat

**Published:** 1877-02

**Authors:** Frank Allport

**Affiliations:** Sycamore, Ill.


### THE CORN SWEAT.

I desire to call the attention of the profession to an old-fashioned, and partially forgotten remedial measure, known as the "corn sweat." In the eruptive, and other fevers, a thorough sweating or sweatings at the very inception of the disease oftentimes will have the effect to cut short the disease, or mitigate its severity. "Cures" where the "Turkish Bath" is administered, show a record in the management of fevers, that testifies abundantly to the efficiency of this plan of treatment. In private practice, this remedy is seldom given properly, and when not is discarded as worthless. The means generally used to bring about a profuse perspiration, are either insufficient, or so complicated or inconvenient as to be seldom brought into use. One of the means usually adopted, is packing in bedclothes and hot bottles or bricks. This is very good, but falls short in a majority of cases, of producing a decided effect. The process I am to describe is very simple. Have the apartment warm; boil fifteen ears of corn in a kettle. Warm five blankets and place them one above another on a bed. Put the patient, undressed, on the blankets and wrap two of them around him, not both at once, but one after the other.

Now quickly dry the boiling ears with a towel, place them all about the patient, and wrap the remaining three blankets one by one around him, well up under the chin. Adults and older children, should be kept in the sweat three-quarters of an hour to an hour. Children of three to six years, half an hour. After profuse perspiration, the whole or a part of the corn may be taken away, after which the blankets may be removed, one by one, at intervals of a few minutes. The last one should remain until the surface of the body is quite dry, after which the patient should be put into a warm bed.

I believe if this plan is strictly followed there is no danger of "taking cold." Care should be taken during the bath that the blankets be well wrapped around the neck. Patients will almost inevitably endeavor to get some air from this quarter. The bath as thus administered has been extremely useful in my hands. I had two cases of typhoid fever, one case of congestive chills, a number of cases of ordinary ague, two cases of pneumonia, two cases of dropsy, etc., that were benefitted by the "corn sweat."

FRANK ALLPORT, M.D.

Sycamore, Ill.

